# Surveillance of the Sensitivity towards Antiparasitic Bath-Treatments in the Salmon Louse (*Lepeophtheirus salmonis*)

**DOI:** 10.1371/journal.pone.0149006

**Published:** 2016-02-18

**Authors:** Peder A. Jansen, Randi N. Grøntvedt, Attila Tarpai, Kari O. Helgesen, Tor Einar Horsberg

**Affiliations:** 1 Norwegian Veterinary Institute, Oslo, Norway; 2 NMBU School of Veterinary Science, Sea Lice Research Centre, Oslo, Norway; 3 Sea Lice Research Centre, Department of Biology, University of Bergen, Bergen, Norway; Institute of Marine Research, NORWAY

## Abstract

The evolution of drug resistant parasitic sea lice is of major concern to the salmon farming industry worldwide and challenges sustainable growth of this enterprise. To assess current status and development of *L*. *salmonis* sensitivity towards different pesticides used for parasite control in Norwegian salmon farming, a national surveillance programme was implemented in 2013. The programme aims to summarize data on the use of different pesticides applied to control *L*. *salmonis* and to test *L*. *salmonis* sensitivity to different pesticides in farms along the Norwegian coast. Here we analyse two years of test-data from biological assays designed to detect sensitivity-levels towards the pesticides azamethiphos and deltamethrin, both among the most common pesticides used in bath-treatments of farmed salmon in Norway in later years. The focus of the analysis is on how different variables predict the binomial outcome of the bioassay tests, being whether *L*. *salmonis* are immobilized/die or survive pesticide exposure. We found that local kernel densities of bath treatments, along with a spatial geographic index of test-farm locations, were significant predictors of the binomial outcome of the tests. Furthermore, the probability of *L*. *salmonis* being immobilized/dead after test-exposure was reduced by odds-ratios of 0.60 (95% CI: 0.42–0.86) for 2014 compared to 2013 and 0.39 (95% CI: 0.36–0.42) for low concentration compared to high concentration exposure. There were also significant but more marginal effects of parasite gender and developmental stage, and a relatively large random effect of test-farm. We conclude that the present data support an association between local intensities of bath treatments along the coast and the outcome of bioassay tests where salmon lice are exposed to azamethiphos or deltamethrin. Furthermore, there is a predictable structure of *L*. *salmonis* phenotypes along the coast in the data, characterized by high susceptibility to pesticides in the far north and far south, but low susceptibility in mid Norway. The study emphasizes the need to address local susceptibility to pesticides and the need for restrictive use of pesticides to preserve treatment efficacy.

## Introduction

Salmon farming has grown to become a large and economically prosperous international industry over the last decades. Norway holds a leading position as a producer of farmed salmonids with an annual production of about 1.2 million tonnes, which is roughly half of the worldwide production[1]. Due to the economic success of salmon farming, Norwegian policy makers have recently launched a plan for future sustainable growth of this industry [1]. There are, however, obstacles to overcome to ensure sustainability of such growth, especially with regard to the spread of pathogens. The marine phase of salmon farming is conducted in open water net pens allowing free water exchange with the surrounding environment. This open form of production implies that pathogens may be transmitted between neighboring farms. Farm to farm distances, as well as hydrodynamic water contact between farms, have been reported to play a major part in the transmission of many economically important diseases in salmon farming [2–4]. Between farm distances have also been reported to predict sea lice (Copepoda, Caligidae) transmission between farms both in Norway and Chile [5–7]. This emphasizes host-pathogen density dependent effects on pathogen transmission. Hence, all else being equal, increasing local densities of farmed salmon will increase the risk of pathogen infection.

In the major salmon farming areas around the world, sea lice infestation levels are regulated by setting maximum thresholds to legal levels of sea lice abundances on farmed fish. To keep within the legal levels of infestation, antiparasitic drugs have been and are at present indispensable, even though an increasing focus is on non-medical control methods such as the use of cleaner fish [8, 9]. A strategy to control sea lice that involves chemotherapy, however, relies on effective pesticides being available. This situation is now compromised by reduced sensitivity or resistance in sea lice towards a variety of different pesticides [10–15]. Among factors that drive the evolution of pesticide resistance are the intensity and frequency of treatments with a particular pesticide, and the population size of sea lice that are exposed to treatments as opposed to those not exposed [16, 17]. Increased rates of sea lice transmission in areas with high local biomasses of farmed fish are accompanied by increased rates of pesticide treatments to control sea lice infestations [5]. Hence, increasing local densities of fish will imply treatment of more sea lice, with a higher frequency of treatments and possibly with increasing dosages of pesticides to achieve acceptable effects, which all may contribute to intensify the selection of resistannce to chemotherapy in pathogens [11, 16, 18]. The evolution of pesticide resistant parasitic sea lice is thus of major concern to the salmon farming industry and challenges the prospect of sustainable growth of this enterprise in Norway.

In order to follow the current status of the sea louse *Lepeophtheirus salmonis’* (also called the “salmon louse”) sensitivity towards different pesticides used for parasite control in Norwegian salmon farming, a national surveillance programme was implemented by the Norwegian Food Safety Authority in 2013 [19]. The programme aims to summarize data on the use of different pesticides to control *L*. *salmonis* and to test *L*. *salmonis* from farms along the Norwegian coast for sensitivity towards pesticide exposure in biological assays (bioassays). In the present paper we analyse two years of bioassay data for bath-treatment pesticides from this surveillance programme. The focus of the analysis is to what extent explanatory variables predict whether *L*. *salmonis* are immobilized/die or survive pesticide exposure in the bioassays, with special emphasis on testing spatial associations between *L*. *salmonis* sensitivity and local treatment intensity, or geographic location of test-farms along the Norwegian coast.

## Materials and Methods

Drugs used in the bioassays were only used to expose salmon lice, not fish. Therefore no permits or ethical approvals were needed to be obtained for the surveillance programme.

### Bioassays

Professional fish health personnel from 11 fish health services along the Norwegian coast were engaged to perform the bioassays on *L*. *salmonis* from different salmon farms (test-farms). The test-farms within the preselected districts from which the samples were collected were selected by the fish health personnel. Test-farms volunteered to participate in the surveillance programme on the conditions i) that they were informed about the test-results, and ii) that farm identities were not made public. The test personnel were given a test protocol, identical stock solutions of the different pesticide agents and standard equipment to standardise test conditions.

Two dose bioassays with 24 hours exposure to the chemical treatment agents, using low and high concentrations of either deltamethrin (low = 0.2 μgL^-1^ (ppb) and high = 1 μgL^-1^, respectively) or azamethiphos (low = 0.4 μgL^-1^ and high = 2 μgL^-1^, respectively), were conducted according to the detailed descriptions in Helgesen & Horsberg [20]. For each farm series of tests, a control test, with no active substance, was also conducted. This was done to assess reasons for mortality not being due to exposure to active substance, with the possibility to discard farm bioassays where the control group mortality was in excess of 20% [20]. Control mortality was in no case > 20%, so no tests were discarded due to high control mortality.

The outcomes of a given test were registrations of individually tested *L*. *salmonis* as immobilised/dead (dead or immobilised when the results were evaluated), as opposed to live. *L*. *salmonis* included in a given test series were identified with respect to developmental stage (preadult I or II or adult) and gender (male or female).

Table 1 summarizes the number of farms tested with bioassays and the number of *L*. *salmonis* that were exposed to the different pesticides in 2013 and 2014 in the bioassays. With the exception of one farm tested with deltamethrin in March 2014, all tests were performed in the autumn (September–November in 2013; July–November in 2014).

**Table 1:** The number of farms tested with bioassays, the number of salmon lice (npar) and the range of salmon lice per farm (f^-1^) exposed to the antiparasitics.

	2013			2014		
Antiparasitic	farms	npar	range f^-1^	farms	npar	range f^-1^
Azamethiphos	48	3716	22–219	58	3750	26–128
Deltamethrin	56	4159	24–222	74	4881	24–159

### Salmon farming data

Farmed salmonids are exposed to *L*. *salmonis* infestations in the marine phase of the production. The focus of the national surveillance programme on *L*. *salmonis* sensitivity towards pesticides is therefore on salmon farms in the marine environment. Norwegian salmon farms are registered with an identifying location number and a geo-reference in the Aquaculture register, which is operated by The Directorate of Fisheries (http://www.fiskeridir.no/English). Marine salmon farms are required to report counts of *L*. *salmonis* and whether antiparasitic bath or in-feed treatments have been applied, on a weekly basis.

#### Treatments and treatment kernel densities

To test if the outcomes of the bioassays were associated with the number of farm treatments with similar pesticides, or the local densities of such treatments, all farm-associated reports of bath-treatments in 2013 and 2014 were compiled. Bath-treatments to control *L*. *salmonis* are reported according to different categories of substances, including azamethiphos, deltamethrin, cypermethrin, hydrogen peroxide, or as “other”. Reports of the use of hydrogen peroxide were excluded from the compiled bath-treatments. Furthermore, we pooled deltamethrin and cypermethrin in the treatment category pyrethroids in the following.

Combining the substances azamethiphos and pyrethroids in bath-treatments has become an increasingly widespread practice in Norwegian salmon farming (Sevatdal 2015, http://www.fhf.no/prosjektdetaljer/?projectNumber=900838, accessed November 16^th^ 2015). Since there is no reporting category that specifically covers combined treatments, we assume that the “other” category represents such treatments. Table 2 summarizes the number of unique marine salmon farms that reported bath-treatments and the total number of reports for the different treatment categories, in 2013 and 2014.

**Table 2:** The total number of farm-reports for each bath treatment category for the years 2013 and 2014. The number of different farms that reported bath treatments were 474 (mean = 2.02 and max = 7 treatments per farm) in 2013 and 470 (mean = 2.34 and max = 8 treatments per farm) in 2014.

Treatment category	Reported treatments	
	2013	2014
Azamethiphos	486	568
Pyrethroids	1006	771
Others	324	540

When treating a farm with a bath-treatment, it is in general mandatory to treat all pens on the farm (https://lovdata.no/dokument/SF/forskrift/2012-12-05-1140, accessed November 16^th^ 2015). Bath treatments of single farms may take weeks in order to finish all pens. A single treatment may therefore be reported over consecutive weeks. To summarize the accumulated number of bath-treatments per farm per year, we regarded sequences of treatments over consecutive weeks to count for one treatment (Table 2). The longest sequence of weekly treatment-reports, accounting for 1 farm treatment, extended over 4 weeks. The number of treatments for the given years and farms where bioassay tests were conducted was extracted from the summary number of treatments (Table 2) and are given in the supporting information data file as the variable “Farm bath-treatments”, S1 Dataset.

The numbers of bath-treatments per farm per year were used to calculate spatial kernel densities of bath-treatments. For each of the years 2013–2014 all treatment reporting farms were projected onto a map in ArcView (ESRI, Redlands, CA, USA). Spatial kernel densities of treatment were then calculated for the number of treatments per farm, using the Kernel Density tool in the ArcView extension Spatial Analyst (ESRI, Redlands, CA, USA), a grid size of one by one km and radii of 25 km (KD1) or 50 km (KD2). The KD1 and KD2 quantities for the farms that were tested with bioassays were extracted by the “Extract values to points” tool in Spatial Analyst (Table 3). KD1 for test-farms in the test-year is given in the supporting information, S1 Dataset.

**Table 3:** Descriptive statistics for the variables predicting the binomial outcome: dead or live salmon lice in bioassays with exposure to azamethiphos or deltamethrin. ΔAIC represents the difference in AIC values when omitting the given variable from the final regression model (Table 4) with an AIC of 17889 (not given for Farm bath-treatments since this was not part of the final model).

Variables	Mean (q 0.1–0.9)/count	Dead	Live	ΔAIC
(factor levels)				
Kernel density (KD1) ^1^	0.021 (0.008–0.035)	0.018	0.022	95
Farm bath-treatments^2^	2.41 (0–5)	2.17	2.54	
Geoindex^3^	8.58 (0.51–18.17)			100
Year 2013	7875	3127	4748	83
Year 2014	8631	2899	5732	
Azamethiphos	7466	2803	4663	136
Deltamethrin	9040	3223	5817	
Male	7512	2913	4599	79
Female	8994	3113	5881	
Adult	12105	4495	7610	84
Preadult 1	1203	455	748	
Preadult 2	3198	1076	2122	
High concentration	8359	3741	4618	786
Low concentration	8147	2285	5862	

^1^ was entered square root transformed and centered in the regression model (Table 4).

^2^was entered square root transformed (Table 4).

^3^was entered squared and scaled in the final regression model (Table 4).

#### Geoindex

**Fig 1:**
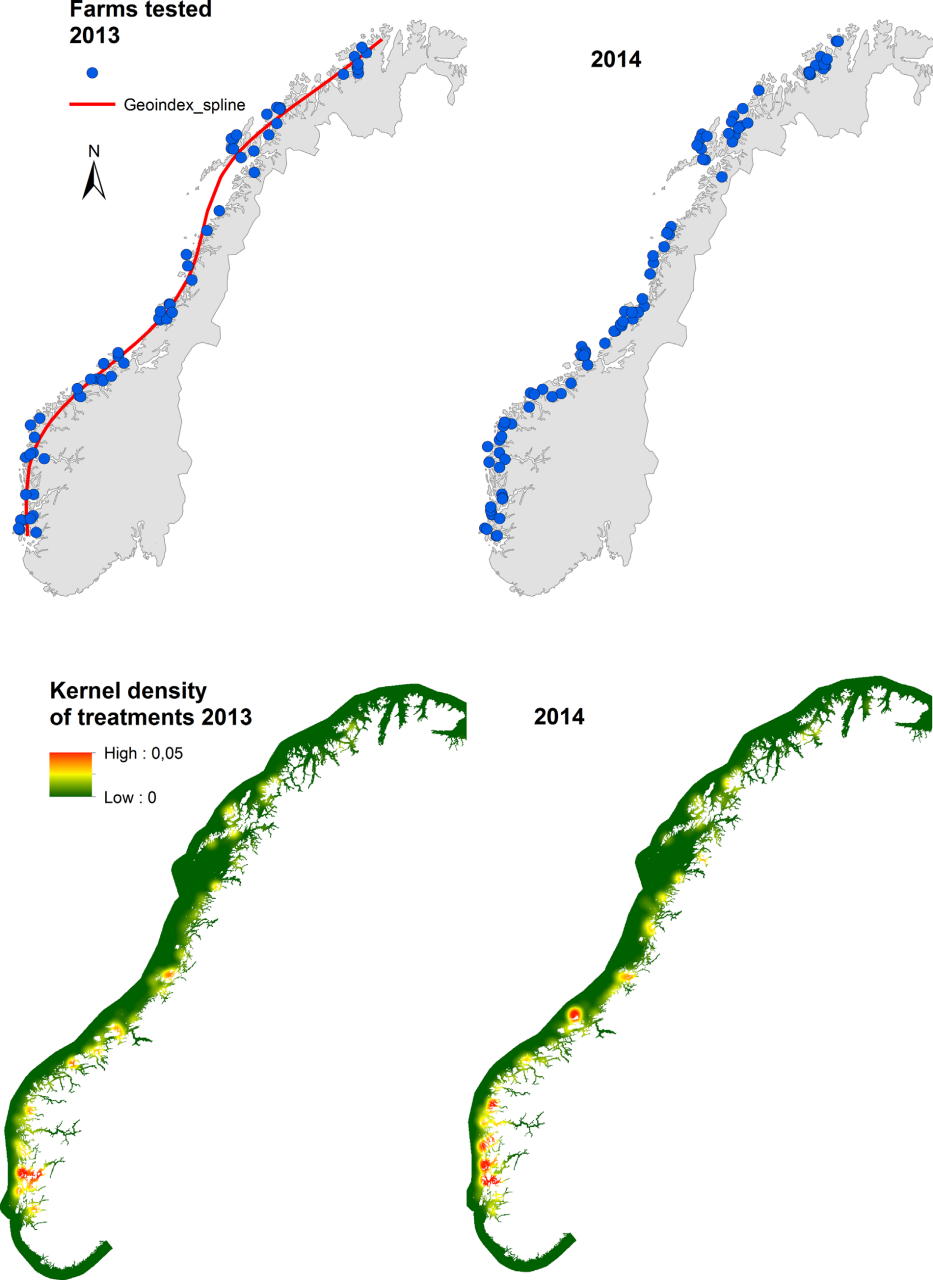
Farms tested with bioassays in 2013 and 2014 (upper panel) and kernel densities of bath-treatments against salmon lice along the Norwegian coast in 2013 and 2014 (lower panel). The red line in the upper left panel shows the plot of the geoindex regression spline through farm latitudes and longitudes (see *2*.*1*.*2 Geoindex*).

To test if there was a spatial effect of location along the Norwegian coast on the outcome of the bioassays, we constructed a geoindex for all test-farms accounting for both longitudes and latitudes using the method presented in Kristoffersen et al. [21]. This geoindex first orders the farms in a south to north gradient and additionally account for their east to west gradient. This was performed using a local polynomial regression (loess; [22]), where the dependent variable comprised of farm latitudes and the independent variable of farm longitudes (Fig 1). Each farm was then projected onto the regression line and attributed a geoindex according to the expression given in Kristoffersen et al. (p. 138)[21].

The coordinate system used for all calculations and displayed in figures was WGS84 projected on to zone N33, using decimal degrees.

##### Additional variables

Additional variables that were used to predict the outcome of bioassays were the factors: year (2013/2014); pesticide agent (azamethiphos/deltamethrin); concentration (high/low); developmental stage (preadult I, preadult II, adult) and gender (female/male) of *L*. *salmonis*.

### Statistical approach

The data were analysed on the level of individual *L*. *salmonis* using the binomial outcome immobilised/dead (1) or alive (0) after bioassay exposure, as the dependent variable in logistic regression. Due to the hierarchical nature of the data, with individual *L*. *salmonis* being nested in tests from given farms, we used a mixed effects model approach, with unique farms tested in a given year as a random variable. We separated between years because ten farms were tested both in 2013 and in 2014. The total number of farms tested was 60 in 2013 and 79 in 2014, giving a total of 139 unique farm-years. Model selection was performed backwards, starting with a model including all the variables listed in Table 2. The criterion for excluding variables was that exclusion reduced the Akaike’s Information Criterion (AIC) value when models were compared. The only variable that was excluded after this criterion was Farm bath-treatments (Table 3).

Two different measures of kernel densities of treatments were tested in the model, where the one resulting in the lowest AIC value (KD1) was kept in the final model. KD1 was entered as a square root and centred variable in the mixed effects model.

The geoindex variable was entered as a squared and scaled term in the mixed effects model. Exploring the data using generalized additive models showed a non-linear association between the geoindex variable and the response variable, which was well fitted to a squared geoindex term.

**Fig 2:**
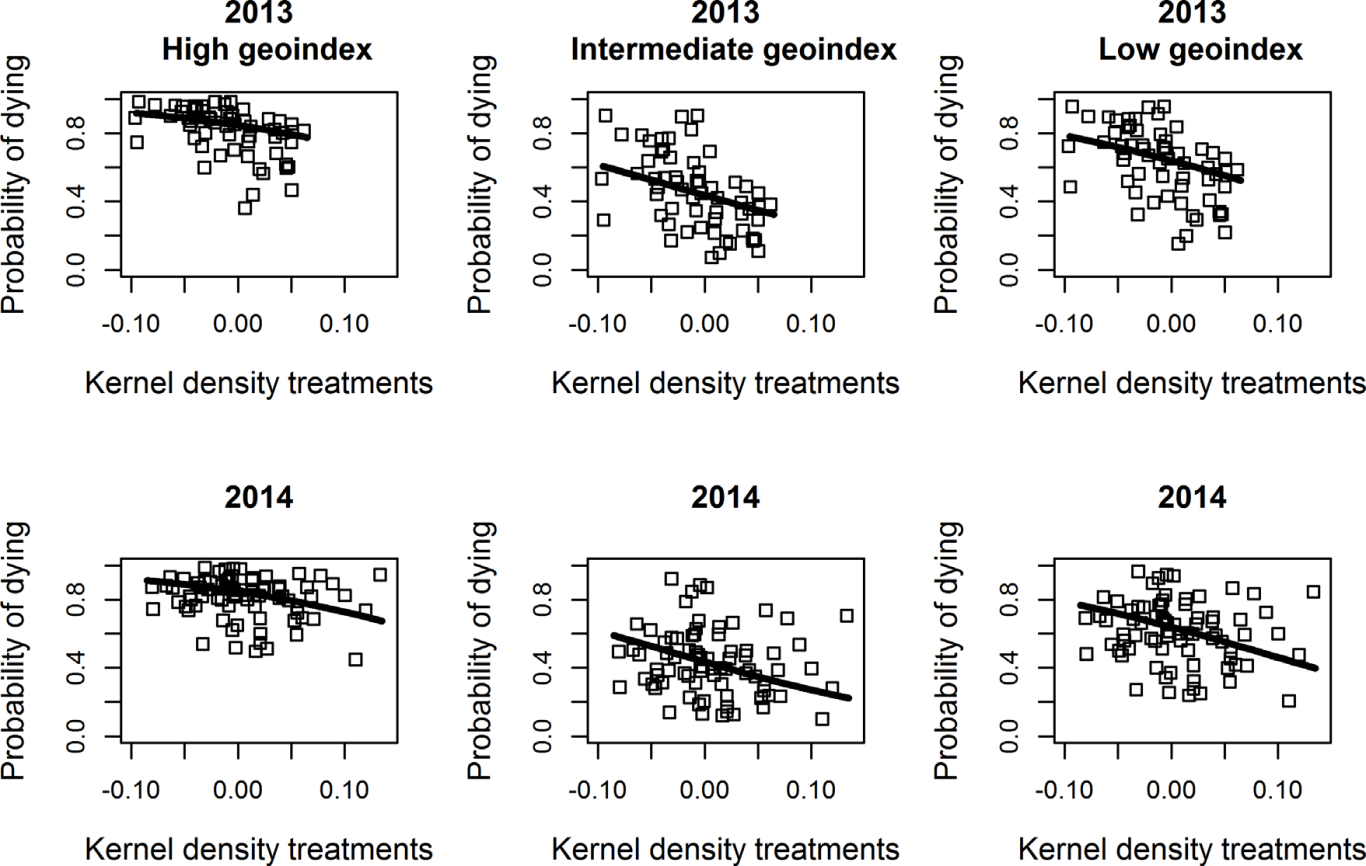
Model predictions of the probability of adult female salmon lice being immobilized/dying when exposed to high concentration azamethiphos in bioassays, as a function of the kernel density of treatments (KD1) attributed to the test farms. The lines represent predictions using only fixed effects, whereas the points represent farm specific predictions when the random effects were accounted for. The upper lower panels represent predictions and farms sampled in 2013 and 2014, respectively. The geoindex scenarios represent predictions assuming farm locations at the 95% high percentile geoindex of 22.4 (High geoindex), the 5% low percentile geoindex of 0.4 (Low geoindex), or a geoindex of 10.3 (Intermediate geoindex). These geoindexes corresponded to lattitudes of 70.3, 64.6 and 59.6 decimal degrees north, respectively (see Fig 3).

The final model was used to predict the probability of adult female *L*. *salmonis* being immobilised/dying as a result of exposure to high concentration azamethiphos bioassays, for three different scenarios with regard to spatial location of farms and as a function of the kernel density of treatments surrounding the farms (Fig 2). These estimates were calculated for 2013 and 2014 using the model fixed effects (lines in Fig 2), as well as for individual farms accounting for the random effects (points in Fig 2). *L*. *salmonis* gender was set to females and stage to adults for the estimations.

The data were analysed using the lme4 package in R [23]. Since not all farms were tested with both azamethiphos and deltamethrin, we also analysed a subset of the data including only farms that were tested with both substances (see S1 Table).

## Results

### Descriptive statistics

Fig 1 (upper panel) shows the distribution of test-farms in the surveillance programme for the years 2013 and 2014, covering most of the salmon producing coast in Norway in both years. The lower panel of Fig 1 shows the kernel density of reported bath-treatments along the coast, using a search radius of 25 km (KD1). The maps of treatment densities generally show an increase of areas with high treatment intensity in 2014 compared to 2013, also extending further north in 2014. The mean number of farm bath-treatments was 1.93 (range 0–5) in 2013 and 2.73 (range 0–8) in 2014.

### Mixed effects model

Descriptive statistics for the explanatory variables entered into the mixed logistic model for *L*. *salmonis* mortality in the bioassays, are presented in Table 3. All variables had a significant effect on the outcome of tests at the parasite level, *i*.*e*. if a given parasite was immobilized/died or survived test exposure to the given pesticide agent, when entered singly as a fixed variable into a mixed effects model with farm-year as a random effect. The KD1 measure of treatment density had a lower ΔAIC value in the full multivariable model than the KD2. Hence, the kernel density KD1 was used in all further analyses. The variable Farm bath-treatments was not significant and did not decrease the AIC value in a full mixed effects model with all variables, and was therefore selected out of the final model. Farm bath-treatments was, however, relatively correlated to KD1 (Pearson r = 0.33).

The final multivariable mixed effects model could be expressed as:

logitP(Yi=1|X)=β0+β1sqrt(KD1)+I(β2Geoindex^2)+β3Antiparasitic+β4Concentration+β5year+β6Stage+β7Gender+ωFarm

The different terms and parameter estimates are featured in Tables 3 and 4. The intra-class correlation coefficient (ICC) was calculated according to Dohoo et al. p. 503[24], as:

$$\sigma^{2}{}_{\text{Farm}}/\left(\pi^{2}/3+\sigma^{2}{}_{\text{Farm}}\right)=0.23,\;\left(95\%\;\text{CI}:\;0.19-0.28\right)$$

where σ^2^_Farm_ represents the between farm variance and π^2^/3 represents a fixed variance of a logistic distribution with mean zero. This indicates that approximately 23% of the variance is at the farm level. The ΔAIC value for the full model compared to a model excluding the random effect was 1961.

**Table 4:** Parameter estimates and confidence intervals for the final mixed effects logistic model for lice-level mortality in bioassay tests. AIC in the full model was 17889.

Parameters	Estimate	95% conf. int.	
		Lower	Upper
Intercept	-0.105	-0.406	0.197
Square root (KD1)	-7.278	-11.464	-3.092
Geoindex	0.559	0.420	0.699
Deltamethrin	-0.347	-0.426	-0.268
Low concentration	-0.954	-1.028	-0.879
Year 2014	-0.508	-0.863	-0.153
Preadult 1	-0.058	-0.211	0.095
Preadult 2	-0.267	-0.371	-0.162
Female	-0.156	-0.231	-0.080
Farm random effect (σ^2^_Farm)_	0.98	0.78	1.27

**Fig 3:**
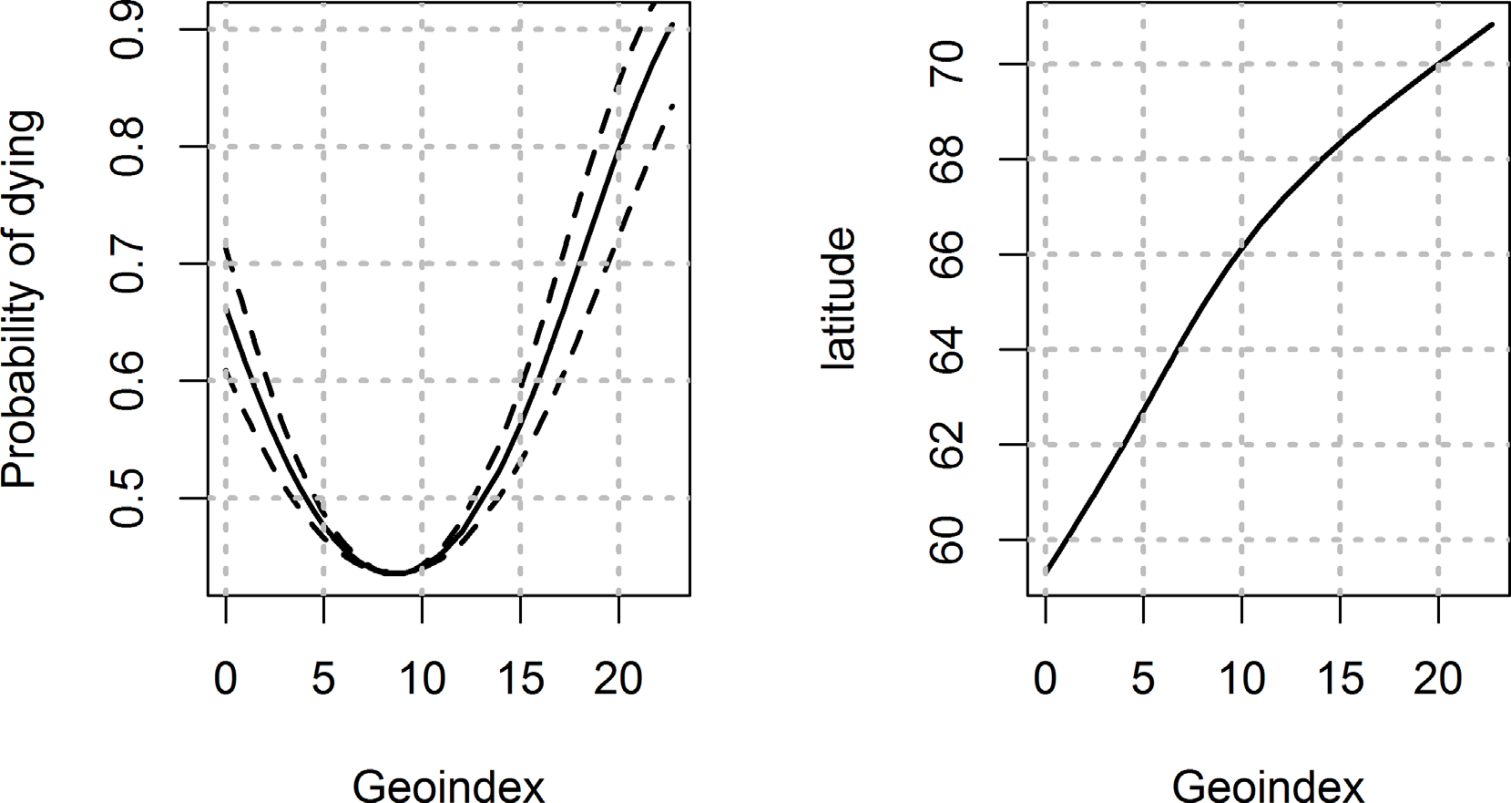
The fixed effect of geoindex (± 95% CI) on the probability of female adult lice dying after exposure to Azamethiphos (concentration = high; year = 2013) in the bioassay tests, for mean values of the kernel density of treatments (KD 1; left panel). The right panel shows the relationship between geoindex and latitudes.

Notable features of the model were also that *L*. *salmonis* mortality varied predictively along the coast. High local densities of treatments were associated with low *L*. *salmonis* mortality in the bioassay tests (Fig 2). Furthermore, the predicted probability of adult female lice dying in high concentration azamethiphos bioassays in 2013 was about 0.9 in the far north, but dropped to below 0.5 at latitudes of about 64–66 decimal degrees north (Fig 3). The model predicted reduced mortalities in 2014 compared to 2013 (odds ratio = exp(-0.508) = 0.60; 95% CI: 0.42–0.86), lower mortalities for deltamethrin compared to azamethiphos (odds ratio = 0.71; 95% CI: 0.65–0.76) and lower mortality for low concentration tests than high concentration tests (odds ratio = 0.39; 95% CI: 0.36–0.42). Effects of gender and developmental stage were less prominent. Taken together, predicted mortality of adult female lice in high concentration tests varied from more than 90% to less than 20% for various combinations of the fixed effects in the model (Fig 2).

**Fig 4:**
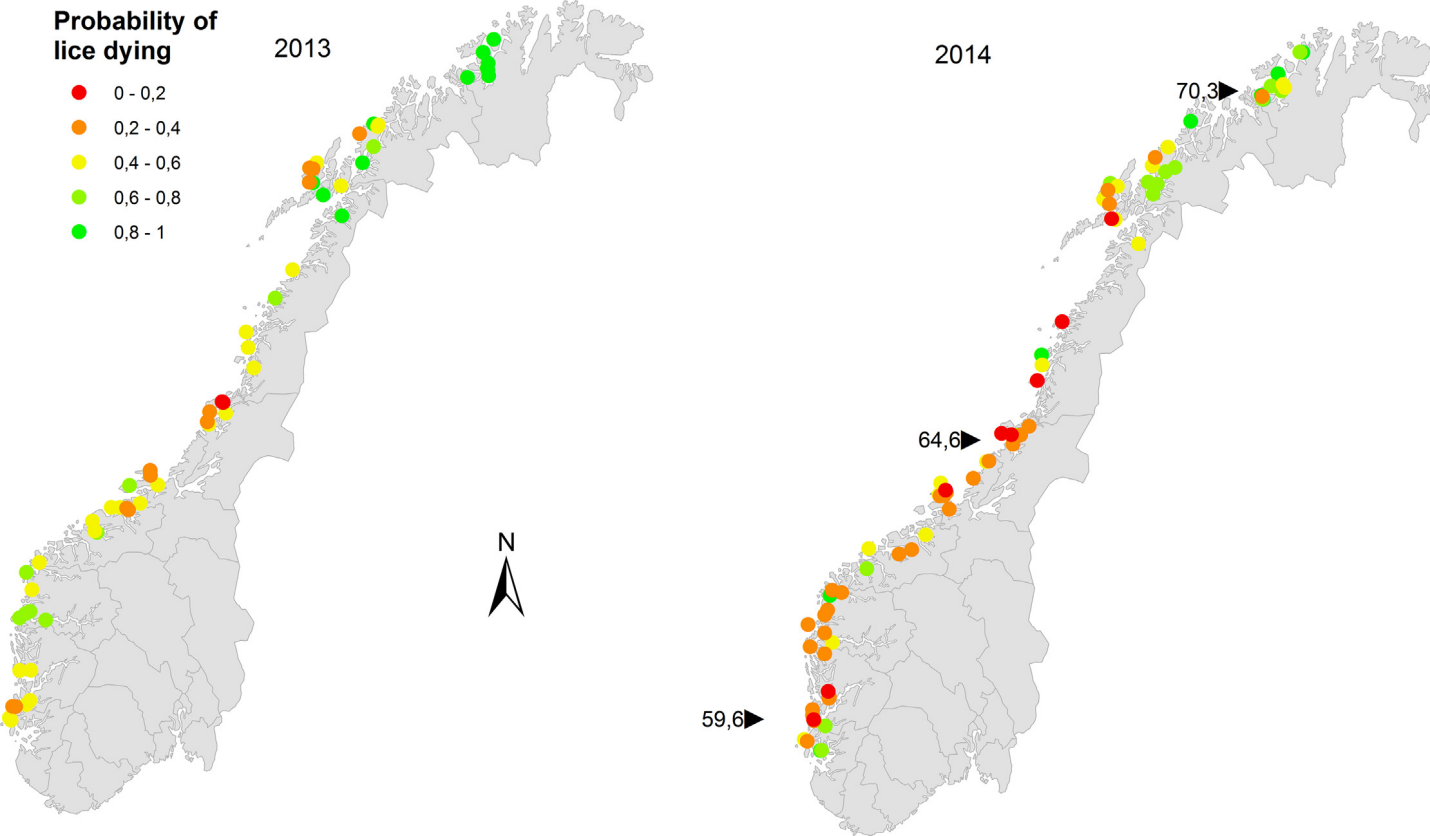
Maps showing farm specific predictions of the probability of adult female lice dying in high concentration azamethiphos bioassays, while accounting for the farm level random effects, in 2013 (left panel) and 2014 (right panel). The black symbols with latitude labels refer to the three modelled scenarios for the geoindex, as shown in Fig 2.

The farm-specific predictions accounting for the random effects showed large variations in the expected outcome of bioassays between farms (Fig 2). In the north, however, the model predicted generally good effect of the test exposures, yielding high *L*. *salmonis* mortality, albeit with marginally reduced mortalities in the north in 2014 compared to 2013 (Fig 4). In general, model predictions were clearly shifted towards lower *L*. *salmonis* mortalities in 2014 compared to 2013 (Fig 4). This is partly due to the factorial effect of year in the model and partly due to the generally higher local densities of treatment in 2014, compared to 2013 (Fig 1).

Fitting the mixed effects model to a subset of the data including only test-farms that were tested with both azamethiphos and deltamethrin did not influence parameter-estimates notably (S1 Table).

## Discussion

The surveillance programme on *L*. *salmonis* sensitivity towards azamethiphos and deltamethrin has so far revealed large variation in the outcome of bioassays, conducted with *L*. *salmonis* sampled from different salmon farms along the Norwegian coast. This large variation was partly explained by fixed predictors and partly related to farm random effects in the analysis of the data, using mixed effects modelling.

Both the local density of treatments (KD1) and the location of test-farms along the Norwegian coast (Geoindex) were significant spatial predictors of the bioassay test outcome, *i*.*e*. whether individual *L*. *salmonis* were immobilised/died or survived pesticide exposure. The effect of local treatments supports a hypothesis linking the frequency of treatments of local *L*. *salmonis* populations with a given pesticide to the development of resistance towards the pesticide in use. Reduced sensitivity and resistance in sea lice to organophosphates and pyrethroids have been reported earlier from many salmon-producing areas [10, 15, 25–27], as well as for *L*. *salmonis* in Norway [28, 29]. Recently, also a specific mutation in an acetylcholinesterase gene in the *L*. *salmonis* genome was identified and associated mechanistically with *L*. *salmonis* resistance to organophosphates [30]. This finding suggests that the phenotypic differences in the susceptibility of *L*. *salmonis* towards pesticide exposure revealed in the present study may be genetically based, and that the phenotypes displaying resistance are promoted by strong selection pressure induced by bath-treatments in salmon farming. Regardless of the mechanisms behind the observed phenotypic differences, however, we believe that the present study is the first to report a large scale spatial association between local treatment intensity and *L*. *salmonis* phenotypes with regard to susceptibility to pesticides. This emphasizes the importance of being restrictive with the use of chemical treatments to control *L*. *salmonis* infestations on farmed salmon, in order to preserve *L*. *salmonis* susceptibility to treatment.

The effect of the spatial geoindex indicates that there is a predictable spatial structure of *L*. *salmonis* phenotypes along the Norwegian coast. With regard to the organophosphate azamethiphos, the mutant *Phe362Tyr* gene coding for acetylcholin esterase was recently found in high proportions in *L*. *salmonis* sampled from farms in mid Norway, but was practically non-existent in *L*. *salmonis* sampled in farms in the far north or far south [31]. This corroborates the findings in the present study, with a spatial distribution of *L*. *salmonis* phenotypes more or less coinciding with that of the mutant *Phe362Tyr* gene which has been associated with azamethiphos resistance [30, 31]. The spatial structure in phenotypes, and at the level of genotypes with regard to the mutant *Phe362Tyr* gene, is remarkable in light of the bulk of previous genetic studies arguing that Atlantic *L*. *salmonis* does not display genetic spatial structuring when analysed using conservative markers such as microsatellites and random SNP’s [32–35]. The uniform structure is probably due to transport over large distances by migrating Atlantic salmon hosts promoting mixing of *L*. *salmonis* from distant areas [35]. This has been taken to emphasize the need for addressing the management of pesticide resistance on an ocean-wide scale rather than on a regional scale [35]. The present findings suggest that a temporal spatial differentiation still may occur. Thus, the management of pesticide resistance in *L*. *salmonis* also needs to address local and regional scales.

For various combinations of the fixed variables in the present mixed effects model, the predicted probabilities of adult female *L*. *salmonis* dying from high concentration pesticide exposure varied from close to 100% to less than 20%. The high-concentration bioassays were designed so as to mimic treatment effects in large scale treatments. If this is the case, then treatment efficacy in most large scale treatments using the present pesticides must be expected to be well below what is considered by the industry to be effective (≥ 90% *L*. *salmonis* mortality; http://lusedata.no/for-naeringen/veileder-for-evaluering-av-behandlingseffekt/, accessed November 16^th^ 2015). Furthermore, given that the high concentration tests approximate treatment effects, then the high random effect at the farm level in the present mixed model should arise from phenotypic traits in the tested *L*. *salmonis*. In this case there must be some factor that is not controlled for in the present data, which contributes to phenotypic variation in pesticide susceptibility in farm populations of *L*. *salmonis*. One such factor could be the presence of the mutation conferring resistance to azamethiphos [30], even though this is not explained by the explanatory variables in the mixed effects model. Alternatively, the farm random effect arises from some un-controlled methodological variation in the bioassays, implying that the high concentration tests are relatively poor predictors of potential treatment effects and hence that the outcome of azamethiphos or pyrethroid bath-treatments in general are hampered by large uncertainties. Development of precise tools to predict the outcome of pesticide treatments of *L*. *salmonis* could have a large potential, not only to ensure acceptable treatment effects for the farmers, but also for the management of pesticide resistance on larger scales [16].

There was a considerable effect of year in the mixed effects model, predicting that *L*. *salmonis* mortalities in 2014 were 0.6 (95% CI: 0.42–0.86) times that of mortalities in 2013, assuming everything else than year to be equal. Whether this is an ongoing trend towards increasing resistance to the present pesticides, remains to be seen. In the present situation, however, where there are limited options with regard to *L*. *salmonis* control [14], along with plans for expanding the production of farmed salmon in Norway [1], it is crusial to follow the development of resistance to applicable pesticides and to gain insight into the mechanisms behind the evolution of pesticide resistance in *L*. *salmonis*. There is limited insight into how selective forces may promote the evolution of pesticide resistance in *L*. *salmonis*; e.g. what is the impact of high rates of treatments with given pesticides, the numbers of pathogens exposed to treatments, or increasing concentrations of pesticide exposure [11, 16, 18]. The same goes for selective forces that may reverse the development of resistance, such as fitness costs associated with resistance traits or effects of mixing of susceptible *L*. *salmonis* into resistant *L*. *salmonis* populations [17, 36]. Insights into these processes are crucial for the opportunity to explore optimal pesticide deployment policies to preserve *L*. *salmonis* susceptibility to pesticides. Optimal pesticide deployment, combined with a growing focus on non-medical control interventions in integrated pest management strategies, should improve the prospects of gaining control with salmon lice infestations in salmon farming. The growing insights into the genetics of resistance to various pesticides provide promising new opportunities to resolve questions regarding the evolution and spread of resistance to chemotherapy in *L*. *salmonis* [11, 30, 35].

## Supporting Information

S1 Dataset Text file comprising the data. (TXT)

S1 Table Mixed effects model statistics for as subset of data. (DOCX)
