# Supplementary material for: Surveillance of the Sensitivity towards Antiparasitic Bath-Treatments in the Salmon Louse (Lepeophtheirus salmonis)
Source: PLoS One. 2016 Feb 18;11(2):e0149006. doi: 10.1371/journal.pone.0149006 (PMC4759459; doi:10.1371/journal.pone.0149006)
Supplement: S1 Table — (DOCX) [file pone.0149006.s002.docx]

Supplementary Table. Final mixed effects logistic model for lice-level mortality in bioassay tests, for the subset of data where both antiparasitic substances were tested at the same time, for given farms. AIC in the model was 15189. The total number of farms and lice included in the subset of data amounted to 92 and 13909, respectively.

| Parameters | |  | 95% conf. int. | |
| --- | --- | --- | --- | --- |
| Key words | Level | Est. | Lower | Upper |
| Intercept (*β*_0_) |  | -0.347 | -0.666 | -0.029 |
| Sqrt (KD1) (*β*_1_) |  | -6.682 | -10.712 | -2.653 |
| Geoindex (*β*_2_) |  | 0.677 | 0.497 | 0.857 |
| Antiparasitic (*β*_3_) | azamethiphos | 0 |  |  |
|  | deltamethrin | -0.353 | -0.432 | -0.273 |
| Concentration (*β*_4_) | high | 0 |  |  |
|  | low | -0.883 | -0.964 | -0.803 |
| Year (*β*_5_) | 2013 | 0 |  |  |
|  | 2014 | -0.403 | -0.770 | -0.036 |
| Stage (*β*_6_) | adult | 0 |  |  |
|  | preadult I | -0.141 | -0.223 | -0.059 |
|  | preadult II | -0.105 | -0.267 | 0.057 |
| Sex (*β*_7_) | male | 0 |  |  |
|  | female | -0.301 | -0.416 | -0.186 |
| Random effects (*ѡ*)  Farm |  | σ^2^_Farm_ |  |  |
|  |  | 0.76 | 0.58 | 1.03 |
